# Data Preprocessing and Model Design for Medicine Problems

**DOI:** 10.1155/2013/625623

**Published:** 2013-03-17

**Authors:** Alberto Guillén, Amaury Lendasse, Guilherme Barreto

**Affiliations:** ^1^Department of Computer Technology and Architecture, University of Granada, Granada, Spain; ^2^Department of Information and Computer Science, Aalto University School of Science, Espoo, Finland; ^3^IKERBASQUE, Basque Foundation for Science, 48011 Bilbao, Spain; ^4^Computational Intelligence Group, Computer Science Faculty, University of the Basque Country, Paseo Manuel Lardizabal 1, Donostia/San Sebastián, Spain; ^5^Department of Teleinformatics Engineering, Federal University of Ceará, Fortaleza, Brazil

Machine-learning disciplines including model design and data preprocessing are crucial in order to obtain a good performance in terms of accurate results and interpretability. However, they are not usually treated simultaneously and, when a model is evaluated, the origin and preprocessing of the data are ignored. Medicine and biomedical research provide a wide variety of problems where machine-learning can be very helpful in decision support, telemedicine, and the discovery of interactions.

These facts motivated the elaboration of this special issue; therefore, it is focused on methods and applications where machine learning could be applied holistically encompassing all stages to solve the problem. The papers included in the special issue go through the intersection between the medical field of application and theoretical models. For example, generalized estimating equations which are a common approach are compared against quadratic inference functions when applied to a lipid and glucose study. It is common in the field of medicine to be suspicious to predictions made by models, so it is interesting also to read another paper presenting the application of machine-learning techniques as a support decision tool that will not replace the expert judgment. This special issue not only considers the application of the models to improve the classification or prediction accuracy but also presents papers where the data are analysed properly. In medicine problems, it is quite common to have continuous and discrete variables in order to show how to deal with these situations; the paper entitled “*Let continuous outcome variables remain continuous*” shows how to apply a popular regression method without dichotomising the variables as this procedure could end up in the lost information.

We hope that the reading of this special issue will help medicine researchers to be aware of new methods and machine-learning techniques as well as to see how they could be applied. We also hope that the machine learning community can see here a wide variety of problems where the models and algorithms they create could be applied providing useful results.

*Alberto Guillén* *Amaury Lendasse* *Guilherme Barreto*

